# Aryl-Alkyl-Lysines: Agents That Kill Planktonic Cells, Persister Cells, Biofilms of MRSA and Protect Mice from Skin-Infection

**DOI:** 10.1371/journal.pone.0144094

**Published:** 2015-12-15

**Authors:** Chandradhish Ghosh, Goutham B. Manjunath, Mohini M. Konai, Divakara S. S. M. Uppu, Jiaul Hoque, Krishnamoorthy Paramanandham, Bibek R. Shome, Jayanta Haldar

**Affiliations:** 1 Chemical Biology and Medicinal Chemistry Laboratory, New Chemistry Unit, Jawaharlal Nehru Centre for Advanced Scientific Research, Jakkur, Bengaluru, 560064, Karnataka, India; 2 National Institute of Veterinary Epidemiology and Disease Informatics (NIVEDI) Ramagondanahalli, Yelahanka, Bengaluru, Karnataka, 560064, India; Purdue University, UNITED STATES

## Abstract

Development of synthetic strategies to combat Staphylococcal infections, especially those caused by methicillin resistant *Staphyloccus aureus* (MRSA), needs immediate attention. In this manuscript we report the ability of aryl-alkyl-lysines, simple membrane active small molecules, to treat infections caused by planktonic cells, persister cells and biofilms of MRSA. A representative compound, NCK-10, did not induce development of resistance in planktonic cells in multiple passages and retained activity in varying environments of pH and salinity. At low concentrations the compound was able to depolarize and permeabilize the membranes of *S*. *aureus* persister cells rapidly. Treatment with the compound not only eradicated pre-formed MRSA biofilms, but also brought down viable counts in bacterial biofilms. In a murine model of MRSA skin infection, the compound was more effective than fusidic acid in bringing down the bacterial burden. Overall, this class of molecules bears potential as antibacterial agents against skin-infections.

## Introduction

Gram-positive infections, especially those caused by *S*. *aureus* are notorious for developing rapid resistance. MRSA alone is responsible for majority of nosocomial (acquired in the hospital) and community acquired (acquired outside hospital) infections.[1] It has been estimated that due to the rapid increase in resistance, even normal infections of MRSA, such as skin infection and wound infection would have to rely on more expensive and more toxic second-line of antibiotics such as daptomycin and clindamycin.[2] Moreover, resistance against all antibiotics that are currently used for treating skin infections caused by MRSA such as fusidic acid, mupirocin, vancomycin, clindamycin and linezolid has been reported.[3–8] If no action is taken against this impending danger, numerous lives are expected to be lost and cost of health care is expected to increase. Although significant research is being conducted towards identifying novel molecules which have potency against MRSA, a long lasting antibiotic with a novel mechanism of action is still elusive.[9]

Natural and synthetic membrane active agents have shown promise as long lasting antibacterial agents and some of them are undergoing clinical trials.[10,11] Several small molecules that target the bacterial membrane have been shown to be effective against MRSA, such as LTX-109,[12,13] brilacidin,[14] binaphthyl based dicationic peptoids,[15] xanthone derivatives,[16] norspermidine derivatives,[17] cationic biocides[18] etc. Among these, only brilacidin (Cellceutix) and LTX-109 (Lytix Biopharma) have moved beyond preclinical stage and are undergoing Phase III and Phase II clinical trials for treating skin infections respectively.

One of the hurdles faced by promising antibacterial agents is eradicating dormant non-dividing cells.[19] Since most of the current antibiotics target specific physiological processes, they require the bacteria to be in an actively dividing state to carry out their functions. Since in the stationary phase, bacteria divides slowly or not at all, higher dose of antibiotic is required for treatment.[20] More importantly, in case of persistent infections, the strategy followed by bacteria to escape antibiotic treatment is down-regulation of their metabolic activity.[21] Such persister cells are often associated with biofilm related infections and are extremely difficult to treat. Development of strategies to counter such cells and biofilms is medically extremely important and require immediate attention.

Herein, we report the activity of aryl-alkyl-lysines, small molecular membrane active agents to treat different forms of Staphylococcal infections such as planktonic cells, stationary phase bacteria, persister cells and biofilms. The ability of the most potent compound to act on membranes of persister cells was also studied. Treatment with the compound did not select a resistant bacteria even in multiple passages. Against a pre-formed MRSA biofilm, the efficacy of the compound was investigated with respect to reduction in biofilm mass, ability to decrease cell viability in biofilms and its eradication using confocal microscopy. Finally, the efficacy of the compound was established in a murine model of skin-infection caused by MRSA.

## Results

### Synthesis

**Fig 1:**
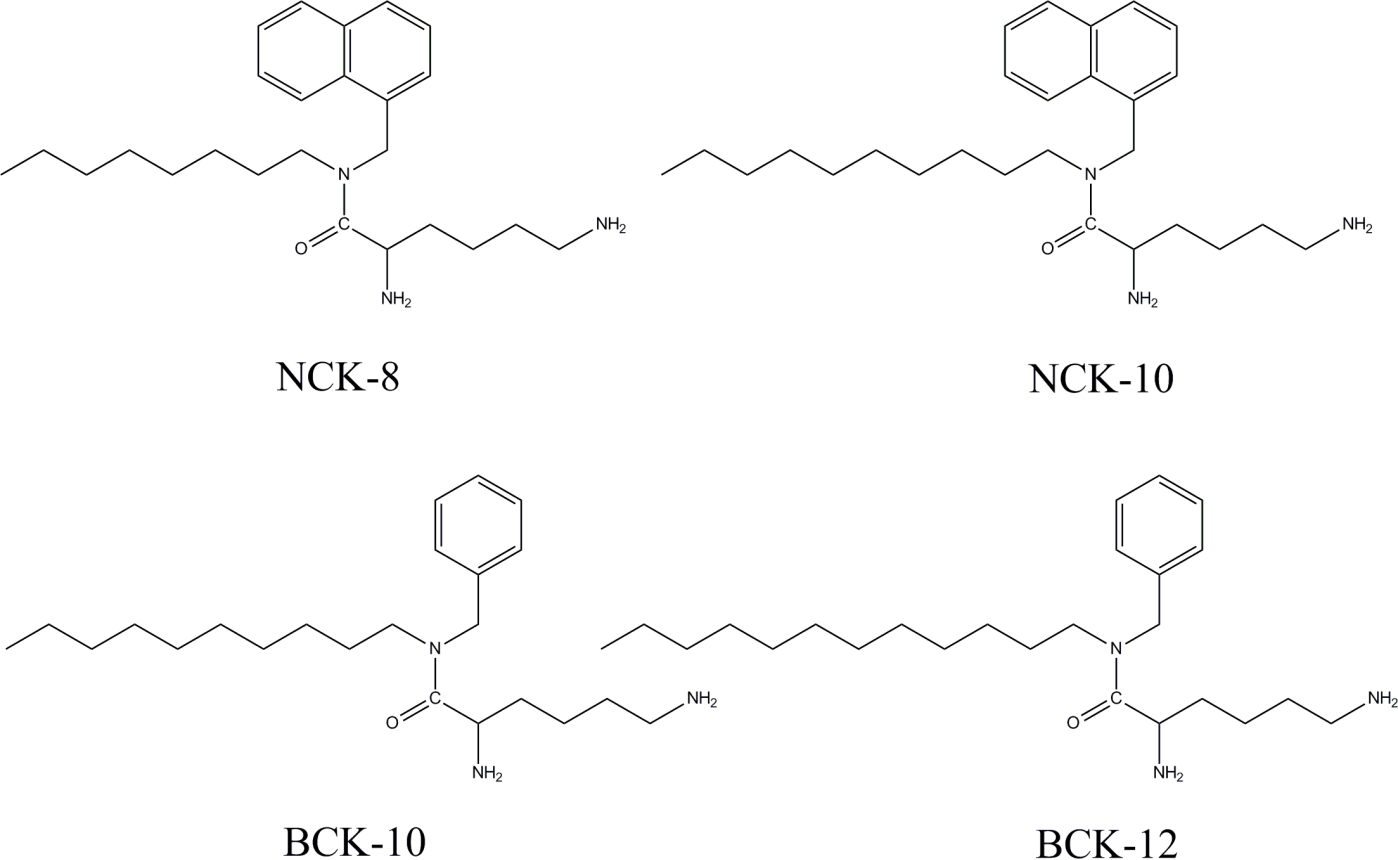
Structures of the compounds used in the study.

The synthesis and characterization of the compounds were reported earlier.[22] In this work four compounds were selected in the study. The compounds were an effective assembly of an aromatic core, a lipid tail and a lysine moiety. Based on the aromatic core and lipid chain they have been named as follows: NCK-8 (naphthalene core with an octyl chain appendage), NCK-10 (naphthalene core with decyl chain appendage), BCK-10 (benzene core with decyl chain appendage) and BCK-12 (benzene core with a dodecyl chain appendage). The structures of the compounds are provided in Fig 1.

### Antibacterial activity

Table 1 lists the activity of the compounds against all the planktonic cells of *S*. *aureus*. In the study four different strains of *S*. *aureus* were used, out of which three of them were resistant to methicillin, with two being clinical isolates from patients of National Institute of Mental Health and Neurosciences, Bangalore. From Table 1 it is clear that NCK-8 and BCK-10, were equally active against *S*. *aureus* (MTCC 737) with a MICs of 10.8 μM and 10.9 μM. Likewise, NCK-10 and BCK-12 were equally active and inhibited *S*. *aureus* growth at around 5.5 μM. Vancomycin against this strain was active at 0.6 μM (as reported earlier).[23] The compounds retained their superior antistaphylococcal activity against MRSA. BCK-10 was active only at 25.5 μM against the ATCC strain of MRSA, while all the others inhibited bacterial growth at less than 10 μM. Against MRSA R3889 and MRSA R3890, which were isolated from two different patients, NCK-10 displayed MICs of 4.4 μM and 4.8 μM respectively. BCK-12 was as active as NCK-10 against these strains with MICs of 4.5 μM and 4.3 μM respectively. Vancomycin was active at 0.6 μM and 0.7 μM respectively (as reported earlier).[24] NCK-8 and BCK-10 too were equally active against both the strains, but their MICs were around two-fold higher than their long chain homologues. Given the structural similarity of the compounds and their comparable antibacterial activity, we chose to carry out the further experiments with a representative compound, NCK-10.

**Table 1:** Activity of compounds against Staphylococcal planktonic cells (including clinical isolates).

Bacterial Strains	Minimum Inhibitory Concentration (μM)			
	NCK-8	NCK-10	BCK-10	BCK-12
*S*. *aureus* MTCC 737	10.8 ± 1.7	5.7 ± 0.5	10.9 ± 0.1	5.5 ± 1.1
MRSA ATCC 33591	8.8 ± 2.6	4.1 ± 0.1	25.5 ± 0.2	5.1 ± 0.6
MRSA R3889	10.8 ± 1.7	4.4 ± 0.3	11.3 ± 1.4	4.5 ± 0.3
MRSA R3890	7.9 ± 1	4.8 ± 0.3	10.2 ± 0.4	4.3 ± 1.1

### Propensity to induce bacterial resistance

**Fig 2:**
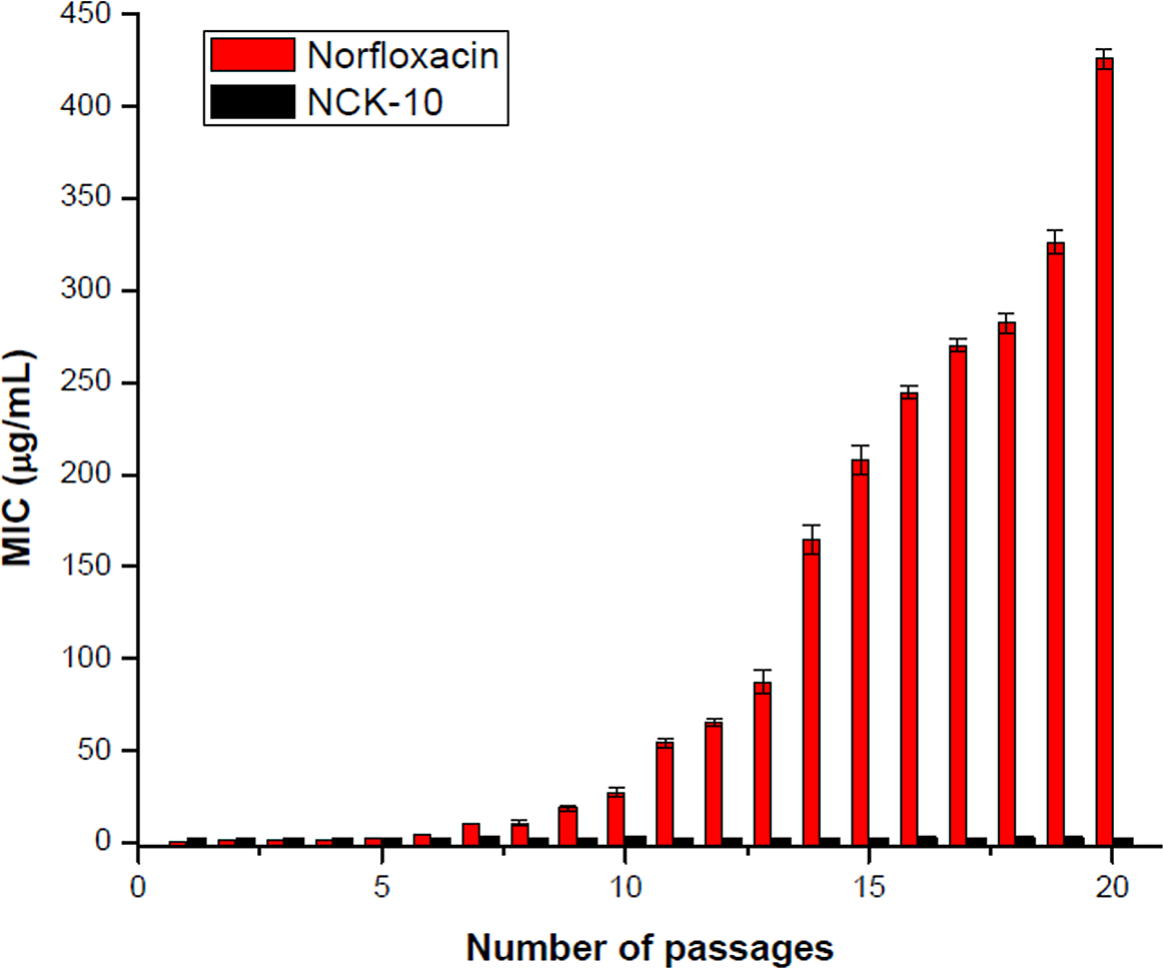
Propensity to induce resistance development in Gram-positive bacteria. Fold of increase in MICs of Norfloxacin and NCK-10 against *S*. *aureus*.

The ability of *S*. *aureus* to develop resistance against NCK-10 was studied to gauge the longevity of this class of compounds (Fig 2). As a positive control for *S*. *aureus*, norfloxacin was used. The MIC of NCK-10 toward *S*. *aureus* (MTCC strain) remained unchanged even after 20 passages, whereas the MIC of norfloxacin increased by 800 fold. This proved that bacteria found it more difficult to develop resistance against NCK-10 in comparison to a clinically used antibiotic norfloxacin.

### Activity at different physiological conditions

**Fig 3:**
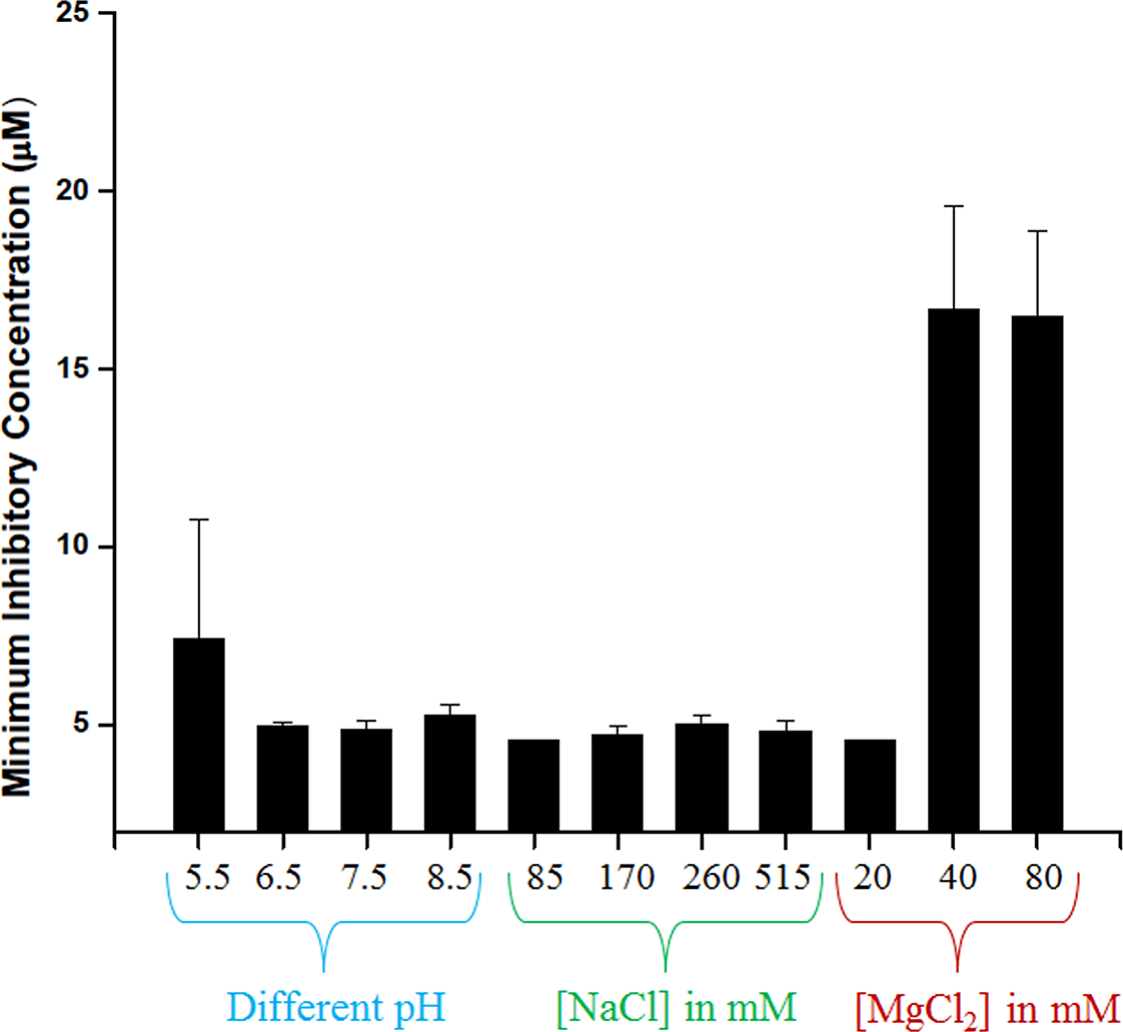
Antibacterial activity of NCK-10 at different physiological conditions.

The antibacterial activity of NCK-10 towards *S*. *aureus* was then studied in varying physiological conditions. This study was expected to provide an idea of the efficacy of the compound for different clinical applications. The study was carried out in varying pH and varying salinity. It is well known that different regions of the body have different pH. Pathogens such as *Mycobacterium tuberculosis*, *Helicobacter pylori* and even *S*. *aureus* can survive at very low pH after colonizing macrophages and gastric lining of the stomach.[25] Moreover, change in vaginal pH also leads to infection.[26] In cystic fibrosis too it is believed that low pH reduces the antimicrobial activity of airway surface liquid.[27] More importantly, the pH of the human skin is acidic (around pH 5.5).[28,29] Thus, in order to gauge the potential of the compounds to treat infections in different regions of the body, it was important to understand its antimicrobial activity in different pH conditions. The antibacterial effect of NCK-10 was first determined in pH ranging from 3.5 to 8.5 (Fig 3). At pH 3.5 and 4.5, almost no growth of *S*. *aureus* was observed. At pH 5.5 NCK-10 showed an activity of 9.4 μM against *S*. *aureus*. At pH 6.5, 7.4 and 8.5, the MIC remained the same at around 5 μM. Likewise, NCK-8, BCK-10 and BCK-12 displayed MICs of 10.8 μM, 10.9 μM and 5.5 μM against *S*. *aureus* at all pH tested (5.5 to 8.5).

Many natural membrane active agents have been reported to lose activity in physiological NaCl concentration of 140 mM.[30,31] Recent evidence suggests that Na^+^ is accumulated at the site of bacterial skin infections.[32] Thus, the efficacy of the compound in presence of varying salinity (percentage of NaCl) was subsequently studied. The concentrations of NaCl (85 mM, 170 mM, 260 mM and 1030 mM) in the growth media was varied first and the efficacy of the compound in the modified growth media was studied. The activity at different concentrations of NaCl is furnished in Fig 3. Against *S*. *aureus* the compound retained its antibacterial activity at all concentrations. Not only NCK-10, the other compounds also retained their activity in these concentrations of NaCl.

On varying the concentration of MgCl_2_ from 20 mM to 80 mM, it was observed that at higher concentrations of the metal, the compound loses activity significantly but no loss of antibacterial activity was observed at 20 mM. Since, the serum concentration of divalent cations like Mg^2+^ are around 3 mM, the compound is expected to work in presence of serum.[33]

### Activity against stationary phase bacteria and persister cells

Antibiotics are known to require very high concentrations to treat stationary phase *S*. *aureus* cells.[20] Remarkably, NCK-10 when treated against *S*. *aureus* cells in stationary phase, showed complete killing at 5 × MIC while ampicillin, which is active at 0.1 μM against planktonic cells of this strain of *S*. *aureus* (MTCC 737), was shown to be inactive at even 50 μM.[34]

**Fig 4:**
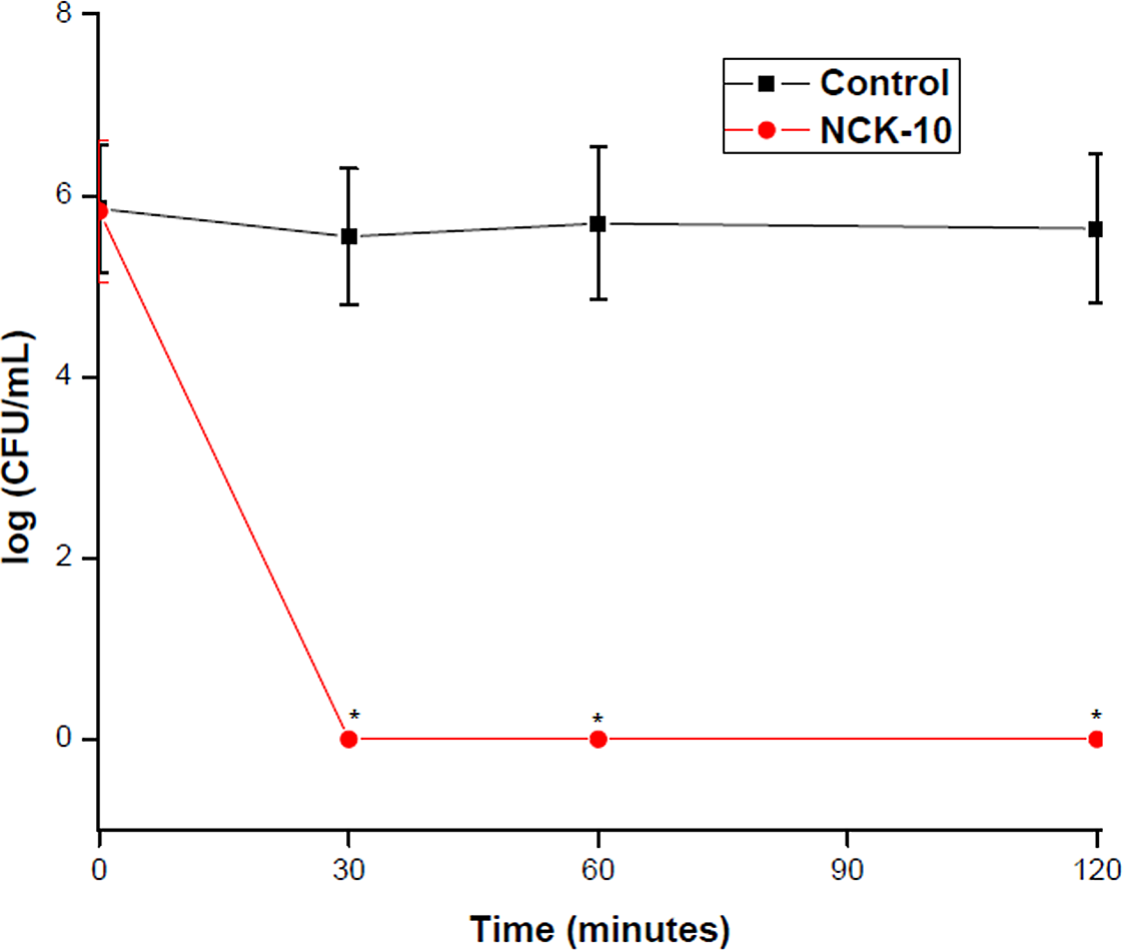
Kinetics of killing of *S*. *aureus* persister cells by NCK-10 at 5 × MIC. (*) indicate that no colony was observed.

Persister cells of *S*. *aureus* are often encountered in biofilm conditions and are known to evade treatment with antibiotics.[35] This is an important problem and there is a requirement for compounds which lyse such cells. As expected ampicillin was not able to inactivate the cells. But NCK-10 at 5 × MIC was sufficient to completely lyse persister cells of *S*. *aureus* in 30 min. (Fig 4) Lower concentrations of NCK-10 did not completely lyse the cells in 2h.

### Mechanism of action

**Fig 5:**
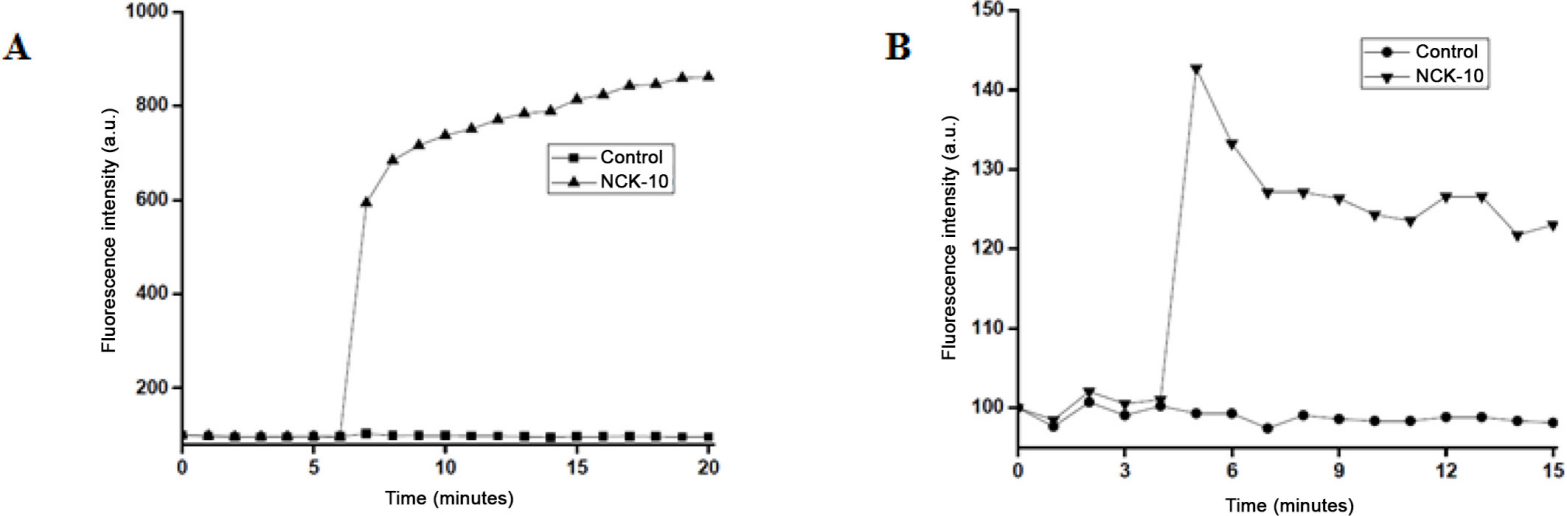
Mechanism of action against persister cells. (A) Depolarization of the membranes of *S*. *aureus* persister cells. (B) Permeabilization of *S*. *aureus* persister cells. The concentration of NCK-10 used is 5 × MIC.

Persister cells, although metabolically different from planktonic cells were hypothesized to remain susceptible to membrane active agents.[21] At 5 × MIC, NCK-10 was able to affect extreme depolarization of the membrane of *S*. *aureus* persister cells within the first minute (Fig 5A). The ability to permeabilize the membrane of *S*. *aureus* persister cells was considerably weaker, as can be observed from variation in fluorescence intensity (Fig 5B).

### Anti-biofilm property of the compounds

**Fig 6:**
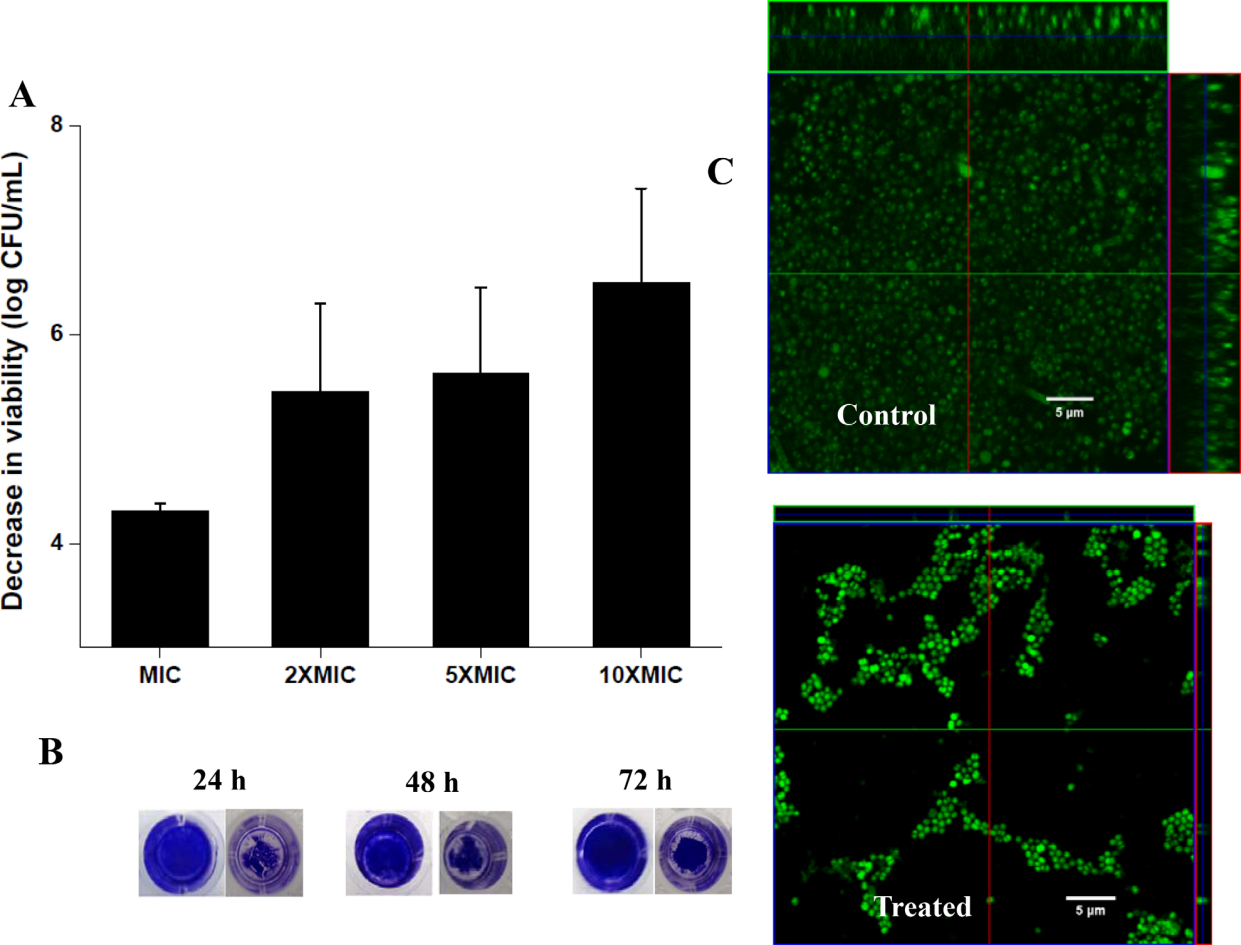
Ability of NCK-10 to disrupt methicillin resistant *S*. *aureus* biofilms. (A) Reduction in viable bacterial count with respect to control at different concentrations of NCK-10. (B) Reduction in biofilm mass by crystal violet staining (Concentration of NCK-10 was 10×MIC). (C) Confocal of image of untreated biofilm and after treatment with NCK-10 (Concentration of NCK-10 was 10×MIC).

*S*. *aureus* biofilms are considered to be serious impediments to healthcare officials all over the world. Agents that can disrupt pre-formed biofilms and kill the bacteria dispersed from biofilms are elusive and still represent an unmet need in healthcare. As can be seen from the Fig 6A, NCK-10 was effective in reducing the number of viable cells of 24h pre-formed MRSA (ATCC 33591) biofilms in a concentration dependent manner. At 10 × MIC, NCK-10 was effective in reducing biofilm mass at 48h and 72h pre-formed biofilms as well (Fig 6B). Confocal images bear a further proof of the anti-biofilm property of the compounds. In the untreated case, there was a thick biofilm which was 23 μm in thickness, while in the treated case, the thickness of the dispersed biofilm was only 2 μm (Fig 6C).

### Acute dermal toxicity

The acute dermal toxicity was performed in accordance with the OECD guidelines. To do the study we used 200 mg/kg of NCK-10 (5 times the concentration used in the subsequent infection study). Careful visual observation of the animals showed that application of the compound caused no irritation, tremors, convulsions, salivation or diarrhea. In fact, steady growth of fur was observed on the mice even after the application of the compound. By the end of seven days, almost complete regeneration fur was observed in all the test animals. No morbidity was observed among the test animals and it was concluded that the compound caused no toxicity to the skin even at concentrations as high as 200 mg/kg.

### *In-vivo* murine model of skin-infection

**Fig 7:**
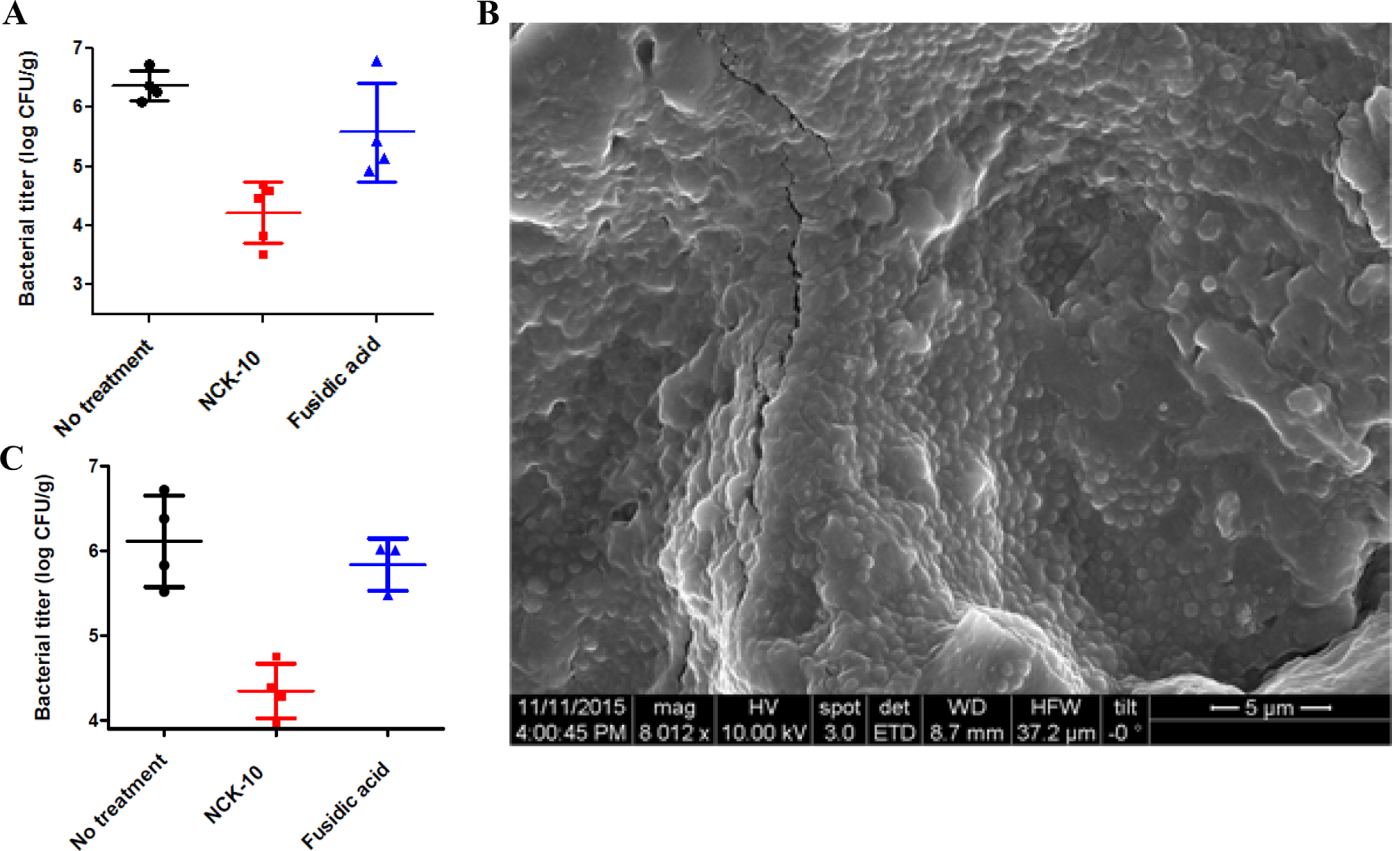
*In-vivo* models of infection. (A) Bacterial titer in MRSA skin infection model (with planktonic cells). (B) Scanning electron micrographs of the wound tissue surface 24 h post inoculation. (C) Bacterial titer in MRSA skin infection model (Biofilm).

*In-vitro* activity of the compounds against MRSA was translated into mice model of skin infection. In order to show the efficacy of the compounds two models of skin infection was used. To do the experiment, a wound was first induced while shaving the back of each mouse until skin tissue was red and glistening. The wounds were then inoculated with approximately 10^7^ CFU of multi drug-resistant methicillin-resistant *Staphylococcus aureus* (MRSA, ATCC 33592). In the first model treatment of the compounds was initiated four hours after infection was set. In this model, NCK-10 or fusidic acid, was dosed at 40 mg/kg (once a day) for seven days by adding a 40 μL droplet (concentration 20 mg/mL) on the infected site. The droplet was gently spread on the entire surface of the wound to avoid any drop from rolling down the sides. The compound was able to bring down bacterial burden significantly (p value 0.00014) in a course of seven days (Fig 7A). In fact the effect of the compound was better than that of the approved drug fusidic acid at the same concentration.

In the second model of skin-infection, treatment was initiated twenty four hours after infection was set. It has been previously reported that within 24 h of infection bacteria form biofilms.[36] To establish the biofilms in our model, we had separately set infection on three mice (as described above), sacrificed them after 24h and analyzed their skin sections under FESEM. As can be seen from Fig 7B, large portions of the tissue surface were covered by dense MRSA biofilms. It can be seen that MRSA had already infected the tissues and in certain regions multiple layers could be seen. This clearly indicated that within 24h, biofilms were formed by MRSA on mice skin.

In the biofilm experiment too, fusidic acid was used as a comparator drug. Both NCK-10 and fusidic acid was dosed at 40 mg/kg (in saline) for 7 days as described above. At the end of seven days, the mice were sacrificed and their wounded skin sections were analyzed. The skin at the region of infection was excised and a count of bacterial colonies were obtained from their homogenates. The results showed that while fusidic acid was not effective in reducing bacterial burden (p value 0.45), the compound was able to bring about significant reduction (p value 0.0013) in bacterial burden (Fig 7C). This result indicated that *in-vivo* the compound was able to act on planktonic cells as well was biofilms of MRSA. The fact that it was much more effective in comparison to the approved drug fusidic acid emphasizes on the clinical potential of the compound.

### Skin histology studies

**Fig 8:**
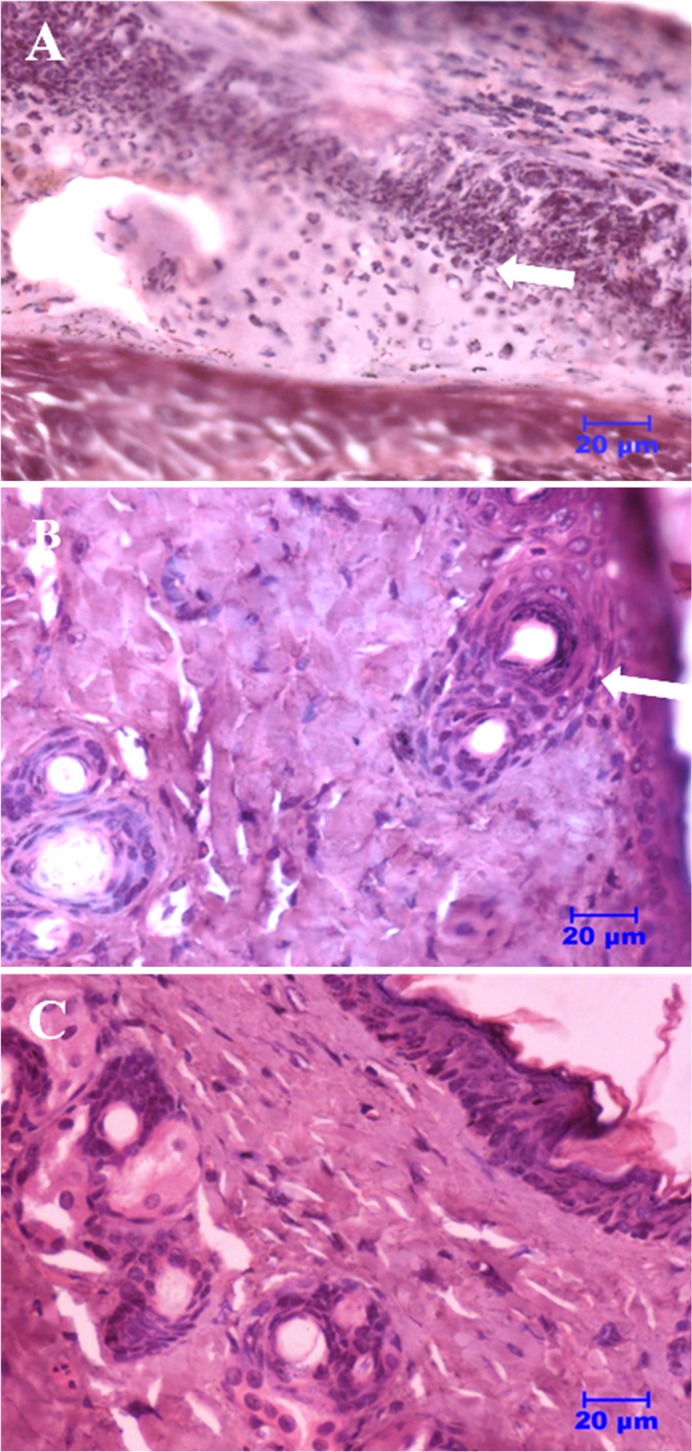
Skin histopathology studies. (A) Untreated (B) Treated with Fusidic acid and (C) Treated with NCK-10.

Skin histopathology studies were conducted to understand the fate of the tissues due to treatment. In order to do this experiment, tissue section from the site of infection was collected from mice belonging to all three groups. These sections were fixed with formalin, embedded in parafilm and stained with hematoxylin and eosin (H & E) and observed under the microscope. The representative figures of different groups of mice are shown in the Fig 8. As can be seen in Fig 8A, tissue section of untreated mice showed severe infiltration of inflammatory cells (mainly neutrophils along with mononuclear cells) attaching to the stratified squamous epithelial cell layer. The arrow points to thick layers of neutrophils and bacterial cells, which are indicated by multilobed nucleus and degenerating cell wall, and bacterial cells with proteinaceous exudates stained pink in colour. Tissue sections of mice treated with NCK-10 showed the presence of stratified squamous epithelial cells with hair follicles (arrow). Presence of sweat glands and sebaceous glands can also be noticed with formation of keratin layer (Fig 8B). No bacterial cells were observed which could adhere to epithelial surface. However, few infiltration of inflammatory cells like neutrophils and mononuclear cells were visible in the subepithelial layer. Tissue sections of mice treated with fusidic acid showed the presence of stratified squamous epithelial cells with sebaceous and sweat glands (Fig 8C). The arrow indicates the presence of hair follicles and keratin layers on epithelial surface. No bacterial cells adhering to epithelial surface could be seen. This experiment proved that the compound, NCK-10 was effective in curing bacterial infection.

## Discussion

Preliminary investigations with aryl-alkyl lysines were reported earlier.[22] The compounds were structurally simple, synthetically easy and showed broad-spectrum potency. Their membrane active nature prompted us to explore their activities further. In this report, we have established the efficacy of the compounds against different manifestations of Staphylococcal infections: planktonic cells, stationary phase cells, persister cells and biofilms.

*S*. *aureus* is known to develop resistance very quickly and resistance has been reported against various classes of drugs including daptomycin (introduced to clinics in 2003).[37] Primarily, development of drugs against MRSA remains a top priority. Against clinical isolates of MRSA, NCK-10 and BCK-12 exhibit extremely potent antibacterial activity. Since the environment of the site of infection might undermine the efficacy of the compound, we determined the activity in different conditions. NCK-10 showed sufficient activity in pH ranging from 5.5 to 8.5. Antimicrobial peptides such as human-β-defensin were shown to be inactivated by high concentration of NaCl.[30] NCK-10 maintained similar potency at high percentages of NaCl against *S*. *aureus*. In presence of higher concentration of divalent cations however, NCK-10 loses activity significantly.

Biofilm related infections are bigger source of worry than infections caused by planktonic cells. Bacterial cells survive within the biofilm at stationary phases and can withstand severe stress to survive for longer periods of time.[21] Higher concentrations of antibiotics are required to eradicate stationary phase bacterial cells which makes such infections extremely difficult to handle. The ability of NCK-10 to kill stationary phase cells of *S*. *aureus* also raises the importance of such membrane active agents against biofilm related infections. Persister cells, on the other hand, are even more notorious as they are known to evade antibiotic treatment by going into dormancy. Such cells pose immense problem in chronic diseases such as cystic fibrosis, endocarditis etc.[19] The remarkable ability of NCK-10 to kill such cells at low concentrations is another reason as to why this class of compounds can have importance in clinics. Although there have been quite a few reports of small molecules active against persister cells, hardly any have probed into their mechanism of action.[38–43] The ability of the compound to act effectively against pre-formed MRSA biofilms is extremely interesting. Since, no dedicated treatment exists against biofilm infections, this result showed that these compounds could be developed as agents for treating both infections caused by planktonic cells as well as those caused by biofilms. Investigations into the mechanism of action of the compound against the persister cells revealed some interesting results. The compounds were able to depolarize and permeabilize the membranes of even *S*. *aureus* persister cells effectively.

Finally, the superior efficacy of the compound was confirmed in murine model of MRSA skin-infection. The problem of MRSA in health care is significant and has been reiterated in the literature. Skin infections caused by MRSA is also a major problem in hospitals. It is also difficult for a practitioner to gauge the exact nature of infections, that is, whether the bacterial cells in the infection is in planktonic condition or have formed biofilms. Since most of the antibiotics do not act against biofilms, treatment of the infection becomes difficult. Since NCK-10 was active both *in vitro* and *in vivo* against planktonic cells as well as biofilms it can be applied irrespective of the stage of infection. Furthermore, it is more efficacious than the approved drug fusidic acid.

Although this report establishes aryl-alkyl-lysines as molecules which bear promise as potent anti-biofilm agents, further studies need to be performed to take them into clinical studies. Future studies with this class of molecules would involve thorough structure-activity relationship study, ability of the compounds to treat systemic infections, their pharmacokinetics and pharmacodynamics, effect of multiple dosage and their effect at different concentrations.

## Conclusion

In conclusion this report effectively documents the range of antistaphylococcal activity of a representative compound of aryl-alkyl-lysines, NCK-10. Due to its membrane active nature, the compound could act against planktonic, stationary phase and persister cells of *S*. *aureus*. The ability of the compound to act on pre-formed biofilms signified the importance of the compounds in chronic infections. In a murine model of MRSA skin infection, the compound was more effective compared to an approved drug fusidic acid in treating infections caused by planktonic cells as well as biofilms of MRSA at concentrations which cause no apparent toxicity to mice. Overall, this initial study has paved the way for development of aryl-alkyl-lysines as promising antibacterial agents against Staphylococcal infections.

## Materials and Methods

### Antimicrobial agents

The synthesis and characterization of the antimicrobial compounds used in this study has been reported previously. Antibiotics used in control study were bought from Sigma-Aldrich.

### Bacterial strains

***S*. *aureus* (MTCC 737)** were obtained from Microbial Type Culture Collection (Chandigarh, India), Methicillin-resistant *S*. *aureus* (MRSA) ATCC 33591 was obtained from the American Type Culture Collection (ATCC). Clinical samples MRSA R3889 and R3990 were obtained from the Department of Neuromicrobiology, National Institute of Mental Health and Neuro Sciences, Hosur Road, Bangalore 560029, India. Bacterial identification was performed by the Vitek 2 Compact 60 system, bioMerieux, France. Culture media and all the antibiotics were from HiMedia and Sigma-Aldrich (India) respectively.

### *In vitro* susceptibility studies

The in vitro susceptibility assay was performed as reported earlier.[22] Staphylococcal strains were grown in nutrient broth at 37°C and subcultured in fresh nutrient broth medium to the desired inoculum concentration and MICs were determined by using broth microdilution method according to CLSI guidelines. (CLSI. Performance Standards for Antimicrobial Susceptibility Testing, M100-S22. Vol. 32 No. 3) Briefly, 150 μL of media containing cells at 10^5^ CFU/mL were added to wells of a 96 well plate containing 50 μL of serially diluted compound. The plate was then incubated at 37°C for 24 h and then the O. D. value was measured at 600 nm using TECAN (Infinite series, M200 pro) Plate Reader. Each concentration had triplicate values and the whole experiment was done at least twice and the MIC value was determined by taking the average of triplicate O. D. values for each concentration and plotting it against concentration. The data was then subjected to sigmoidal fitting. From the curve the MIC value was determined, as the point in the curve where the O. D. is similar to that of control having no bacteria. The results furnished are an average value of at least two independent experiments and each experiment was performed in duplicates/triplicates.

### Propensity to induce resistance development in bacteria

This assay was performed as reported previously.[17] Briefly, MIC values of the compounds (NCK-10 and antibiotics) were determined against *S*. *aureus* (MTCC 737) as described above. After determination of MIC, bacterial cells growing at the highest concentration of the compound (usually half of MIC) were harvested and inoculated into fresh media. This inoculum was subjected to another MIC assay. After 24 h incubation period, cells growing in the highest concentration of the compound from the previous passage were once again harvested and inoculated for another MIC experiment. The process was repeated for 20 passages. The MIC value of the compound was plotted against the number of passages, and the fold increase in MIC was determined. Each MIC experiment was performed in triplicates. The results are furnished as the average of triplicates and indicate the fold of increase in MIC every day.

### Antibacterial activity in different physiological conditions

First the pH and salinity of the culture medium was brought to the desired pH by adding either NaOH (1N) or HCl (1 N) or to the desired salt concentration by adding NaCl and MgCl_2_ to the nutrient agar. The different pH conditions considered were 3.5, 4.5, 5.5, 6.5, 7.4, and 8.5. The different percentages of NaCl considered were 85 mM, 170 mM, 260 mM and 515 mM. Then the antibacterial activity of the compounds was determined in this medium by the same assay as described above. The experiments were performed in triplicates and the result is an average value.

### Activity against Stationary-phase bacteria

This experiment was performed by following a published protocol with little modification. [34] *S*. *aureus* was grown for 6 h in nutrient broth and contained ~10^9^ cfu/mL (determined through dilution plate technique by spread plate method). This was then diluted to 1000 fold and incubated 37°C for 16 h to obtain stationary-phase cultures. At the end of 16h, the cells were centrifuged down, washed twice with Minimum Essential Media (MEM) and resuspended in MEM (GIBCO) media at concentration of 10^6^ CFU/mL. 50 μL of the test compound, NCK-10 or Ampicillin was then added to 150 μL of the stationary-phase bacteria (in wells of a 96 well plate) with the working concentrations of 15 μg/mL and 20 μg/mL respectively. In the negative control the well contained 200 μL of MEM. The 96 well plate was then incubated at 37°C with shaking at 150 rpm. At the end of 2h, 20 μL of aliquots from that solution were serially diluted 10-fold in MEM (GIBCO) media. Then from the dilutions, 20 μL was plated on nutrient agar plates and incubated at 37°C. After 24 h the bacterial colonies were counted and results represented in logarithmic scale, i.e. log (CFU/mL). The results furnished are averages of two independent experiments.

### Isolation of Persister cells

Persister cells of *S*. *aureus* were isolated following a previously published protocol.[34] After growing the respective bacteria to their stationary phases using the protocol mentioned above, *S*. *aureus* cells were then treated with 100 μg/ml ampicillin for 3h. Then they were centrifuged down, and washed with MEM twice and resuspended again in the same media. These cells were then diluted to 10^5^ cells and treated with the compound (at concentrations of 5 × MIC, 2 × MIC and MIC) and also with another round of ampicillin (20 μg/mL) for 2h or left untreated in wells of a 96 well plate as mentioned above. It was then incubated at 37°C with shaking at 150 rpm. At the end of 2h, 20 μL of aliquots from that solution were serially diluted 10-fold in corresponding media. Then from the dilutions, 20 μL was plated on nutrient agar plates and incubated at 37°C. After 24 h the bacterial colonies were counted and results represented in logarithmic scale, i.e. log (CFU/mL). The results furnished are averages of two independent experiments.

### Kinetics of killing of persister cells

*S*. *aureus* persister cells were isolated as described above and diluted to approximately 10^6^ cells. 50 μL of the test compound NCK-10 was then added to 150 μL of the bacterial solution with the working concentration of 5 × MIC. For the control, the same experiment was performed with same volume of MEM instead of the compound. At different time intervals corresponding to 0, 30, 60 and 120, 20 μL of aliquots from that solution were serially diluted 10-fold in 0.9% saline. From the dilutions, 20 μL was plated on nutrient agar plates and incubated at 37°C for 24 h. The bacterial colonies were counted and results were represented in logarithmic scale, i.e., log (CFU/mL). The experiment was performed twice and the results furnished are an average of the two experiments.

### Cytoplasmic membrane depolarization assay

Persister cells were isolated as described above. With these, cytoplasmic membrane depolarization studies were performed as described previously.[18] Briefly, 10^8^ CFU/ml of persister cells were washed and resuspended in 5 mM glucose, 5 mM HEPES buffer and 100 mM KCl solution in 1:1:1 ratio (10^8^ cfu/ml). To this solution DiSC_3_(5) dye (obtained from Sigma-Aldrich) was added to a final concentration of 2 μM. The bacterial suspension containing the dye (200 μL) was preincubated for 20 min in a well of a black 96-well plate with transparent bottom (Dye uptake, and resultant self-quenching). The fluorescence of the bacterial suspension was measured (excitation wavelength: 622 nm; emission wavelength: 670 nm) and allowed to stabilize for 6 min at room temperature before the addition of 2 μL of NCK-10 (final concentration of 5×MIC μM). After addition fluorescence intensity was measured every minute or two for 20 min. The resultant plot was obtained by joining the data points. The experiment was repeated thrice and one representative plot has been furnished.

### Cytoplasmic membrane permeabilization assay

Persister cells were isolated as described above. With these, cytoplasmic membrane permeabilization studies were performed as described previously.[18] To the solution of bacterial cells in 5 mM HEPES and 5 mM glucose (pH 7.2) propidium iodide (PI) dye (obtained from Sigma-Aldrich) was added to a final concentration of 15 μM. The suspension containing the dye (200 μL) was then added to the well of a 96-well plate (black plate, clear bottom with lid) and then after 4 minutes 2μL of NCK-10 was added to the solution to a final concentration of 5 × MIC. Fluorescence intensity was measured at excitation wavelength of 535 nm (slit width: 10 nm) and emission wavelength of 617 nm (slit width: 5 nm). The uptake of PI was measured by the increase in fluorescence of PI for 10 min as a measure of inner membrane permeabilization. The experiment was repeated thrice and one representative plot has been furnished.

### Biofilm disruption assay

The biofilms disruption assays were performed following a previously published protocol.[44] Midlog phase (6 h grown) culture of MRSA were diluted to a concentration of approximately 10^5^ CFU/mL in a nutrient broth supplemented with 1% glucose and 1% NaCl to make the bacterial stock solution. Then 100 μL of this stock were added into 96-well plates (polystyrene microtiter). The plates were then incubated under stationary conditions for 24 h at 37°C. After that medium was removed and planktonic bacteria were washed out with PBS (pH = 7.4) to obtain biofilms at the bottom of the wells. Then, 100 μL of test compound NCK-10 (at MIC, 2 × MIC, 5 × MIC and 10 × MIC) was added to the biofilm and allowed to incubate under stationary conditions for 24 h. One control was made where 100 μL of complete medium were added instead of compound. After 24 h medium was discarded and planktonic cells were removed by washing with PBS. The disrupted biofilms were then scraped with pipette tip following 5 min-exposure to 100 μL trypsin-EDTA (0.25%) (GIBCO). Cell suspension was then vortexed at high speed for 1 min to break up the clumps. The bacterial counts were assessed by plating serial 10-fold dilutions of biofilm cell suspensions on nutrient agar plates. After 24 h of incubation the plates were counted and cell viability was expressed as log (CFU/mL) and compared with non-treated control. The experiment was performed twice.

For visualization the biofilm disruption, 100 μL of 0.1% of crystal violet (CV) was added into the wells and incubated for 10 min at 37°C. Crystal violet solution was then discarded and the plates were washed once with PBS (pH = 7.4) solution. Finally, the stained images were captured using a digital camera.

### Confocal Imaging of Biofilm

Cover slips were first sterilized by soaking them in ethanol followed by drying in flame, and then placing in well of a 6-well plate. Midlog phase (6 h grown) culture of *S*. *aureus* was then diluted to approximately 10^5^ CFU/mL in a nutrient broth supplemented with 1% glucose and 1% NaCl and 2 mL of this stock was added to cover slips containing wells. The plate was then incubated under stationary conditions at 37°C. After 24 h, medium was removed and planktonic bacteria were carefully washed out with PBS (pH = 7.4). Biofilm containing cover slips were then placed into another 6-well plates and 2 mL of test compound NCK-10 (10×MIC) was added to it and allowed to incubate for 24 h. In case of control, 2 mL of complete medium was added instead of compound. At the end of 24 h, medium was removed and planktonic cells were removed by washing with PBS. Coverslips containing biofilm were carefully removed from the well and stained with SYTO-9 (3 μM) and imaged it using a confocal laser-scanning microscope. This experiment was done twice.

### *Invivo* studies

Animal studies were performed according to the protocols approved by Institutional Animal Ethics Committee (IAEC) of National Institute of Veterinary Epidemiology and Disease Informatics (NIVEDI) and Jawaharlal Nehru Centre for Advanced Scientific Research. Acute dermal toxicity studies were performed at Jawaharlal Nehru Centre for Advanced Scientific Research (JNCASR), Bengaluru (CPCSEA/201) in accordance with institutional ethical guidelines. The infection experiments were approved by the Institutional Animal Ethics Committee (IAEC) of National Institute of Veterinary Epidemiology and Disease Informatics (NIVEDI), Bengaluru (881/GO/ac/05/CPCSEA) and carried out as per the guidelines of Committee for the purpose of Supervision and Experiments on Animals (CPCSEA), Ministry of Environment and Forests, New Delhi, India. The mice were housed in individually ventilated cages (IVC) maintained with controlled environment. They were housed in pathogen free conventional caging systems, bedding material used were corncob. The husbandry conditions: Light: dark cycle—12:12 hours, Animal Room Temp: 22 ± 2°C, Relative humidity: 30–40%, Access to feed and water: ad libitum and Water: RO Water. Animals were randomly selected, marked to permit individual identification and kept in their cages for at least 5 days before the experiment to allow for acclimatization to the experimental conditions. Animal handling and experimentation protocols were followed according to OECD Guidelines for the Testing of Chemicals (OECD 425). All care was taken to cause no pain to the animals. Humane endpoints were used to avoid unnecessary distress and suffering in animals following an experimental intervention that would lead to death.

### Acute dermal toxicity

The acute dermal toxicity was performed on five 6 to 8 weeks-old male BALB/c mice (18-22g) following the OECD guidelines. Approximately 24h before the experiment, fur was removed from the back of the mice, first by clipping and then shaving. Care was taken to avoid abrasion of the skin. The area of the shaved portion was roughly 2 cm^2^. NCK-10 was dissolved in saline at concentrations of 100 mg/mL to make working stocks. From this stock, 40 μL was added to the shaved area of the skin to give a concentration of 200 mg/kg per animal. Following the application of the compound, the animals were observed carefully and then observed carefully once every day for 14 days. Particular attention was paid to the changes in fur, eyes and mucous membranes, and observations of tremors, convulsions, salivation, diarrhea, lethargy, sleep and coma.

### MRSA skin infection

The experiment was performed following several previously published protocols with modifications.[8,36,45,46] 6 to 8 weeks-old male BALB/c mice (18-22g) were used for the experiments. The mice were anesthetized by intraperitoneal injection of xylazine-ketamine cocktail. The fur on the back of the mice were then shaved using a sterile razor. The fur was shaved in a manner to induce a wound, that is, reddening and glistening of the exposed skin was observed without bleeding. To this visibly damaged area (~2 cm^2^) of the skin, a bacterial infection was initiated by placing on the skin a 20 μL droplet containing 10^7^ cells of MRSA. This was allowed to dry for 20 minutes and it was ensured that the droplet stayed within the shaved area. In the first experiment one group of mice (n = 5) were treated after 4h with 40 μL of NCK-10 (concentration of 20 mg/mL) at the site of infection (on the shaved area of the skin where bacteria was added). The droplet was gently spread on the entire surface of the wound to avoid any drop from rolling down the sides. Another group of mice (n = 4) were dosed with fusidic acid, a comparator drug, at exactly the same concentration. The dosage for both the compounds were continued for seven days. One group of mice (n = 4) were left untreated and served as a control. 18h after the last dose (to prevent carryover effects) the mice were sacrificed using isofluorane and the infected skin (on the back of the mice) was severed aseptically. The severed part was weighed and placed into about 10 mL of sterile saline and homogenized. The dilutions of the homogenate were plated onto agar plates, which were incubated overnight at about 37°C. The bacterial titer was expressed as log (CFU/g) of weight of the tissue collected and expressed as mean ± S.E.M (standard error of mean).

### Biofilm experiment

For the second experiment, exactly same protocol was followed but the dosage with fusidic acid (n = 3) and NCK-10 (n = 4) was started 24h post infection while in control (n = 7) mice were left untreated. To establish the infection, three mice were inoculated with approximately 10^7^ CFU of bacteria as described above. After 24 h three of these mice were sacrificed using isofluorane. Skin portion from the wound site of the infected mice were severed aseptically, homogenized, serially diluted and plated on agar plates and counted. Small portions of the skin were also imaged under FESEM for visual proof of the formation of biofilm (the experimental protocol is described below). Typically, the count of bacteria 24h after bacterial addition is (6.65±0.03) log.

### FESEM studies

Further, to confirm the biofilm formation, wound tissue surface was imaged by scanning electron microscopy. A piece of skin tissue collected after sacrificing the mice was transferred to buffered 2.5% glutaraldehyde, dehydrated with 50%, 70%, 90%, and 100% ethanol, dried and sputter coated with gold prior to imaging using Quanta 3D FEG, FEI field emission scanning electron microscope.

### Skin histology studies in different groups of mice

The portion of skin was collected from group of mice belonging to untreated control, fusidic acid and NCK-10 respectively and fixed in 10% formalin (10 ml of 40% formaldehyde added to 90 ml of water). The number of sections examined was one per animal and at least three mice per group were used for the study. The tissues were fixed for 48 h and washed for 1 h under running tap water. Then dehydration of the tissues was performed with increasing concentrations of ethanol (70%, 90% and 100% cent; each for 1 h). Then the tissues were washed in xylene for 1 h for two changes. Paraffin embedding was carried by keeping the tissues in melted paraffin at 56°C for three changes. Longitudinal and transverse sections (5μm) were prepared with semiautomatic microtome and placed on glass slide coated with Meyer’s egg albumin. Tissue sections were dried by incubating them for 2 h at 40°C. Rehydration of fixed sections was carried in decreasing grades of alcohol (100%, 90%, 70% and 50%; each for 1 h) and then water. The sections were stained with haematoxylin and eosin stain as per a previously published protocol.[47] Then the sections were covered with DPX (SRL, India) mounting medium with cover glass and observed under light microscope (Nikon, Japan) to study the histopathological changes.
